# Mechanical Properties, Microstructure, and In Vitro Digestion of Transglutaminase-Crosslinked Whey Protein and Potato Protein Hydrolysate Composite Gels

**DOI:** 10.3390/foods12102040

**Published:** 2023-05-18

**Authors:** Haowei Zhang, Juan Wu, Yu Cheng

**Affiliations:** 1 School of Food and Biological Engineering, Jiangsu University, 301 Xuefu Road, Zhenjiang 212013, China; 2212018052@stmail.ujs.edu.cn (H.Z.); wujuan@ujs.edu.cn (J.W.); 2 Institute of Food Physical Processing, Jiangsu University, 301 Xuefu Road, Zhenjiang 212013, China

**Keywords:** potato protein hydrolysate, whey protein, composite gel, texture, microstructure, in vitro digestion

## Abstract

The production of animal protein usually leads to higher carbon emissions than that of plant protein. To reduce carbon emissions, the partial replacement of animal protein with plant protein has attracted extensive attention; however, little is known about using plant protein hydrolysates as a substitute. The potential application of 2 h-alcalase hydrolyzed potato protein hydrolysate (PPH) to displace whey protein isolate (WPI) during gel formation was demonstrated in this study. The effect of the ratios (8/5, 9/4, 10/3, 11/2, 12/1, and 13/0) of WPI to PPH on the mechanical properties, microstructure, and digestibility of composite WPI/PPH gels was investigated. Increasing the WPI ratio could improve the storage modulus (G′) and loss modulus (G″) of composite gels. The springiness of gels with the WPH/PPH ratio of 10/3 and 8/5 was 0.82 and 0.36 times higher than that of the control (WPH/PPH ratio of 13/0) (*p* < 0.05). In contrast, the hardness of the control samples was 1.82 and 2.38 times higher than that of gels with the WPH/PPH ratio of 10/3 and 8/5 (*p* < 0.05). According to the International Organization for Standardization of Dysphagia Diet (IDDSI) testing, the composite gels belonged to food level 4 in the IDDSI framework. This suggested that composite gels could be acceptable to people with swallowing difficulties. Confocal laser scanning microscopy and scanning electron microscopy images illustrated that composite gels with a higher ratio of PPH displayed thicker gel skeletons and porous networks in the matrix. The water-holding capacity and swelling ratio of gels with the WPH/PPH ratio of 8/5 decreased by 12.4% and 40.8% when compared with the control (*p* < 0.05). Analysis of the swelling rate with the power law model indicated that water diffusion in composite gels belonged to non-Fickian transport. The results of amino acid release suggested that PPH improved the digestion of composite gels during the intestinal stage. The free amino group content of gels with the WPH/PPH ratio of 8/5 increased by 29.5% compared with the control (*p* < 0.05). Our results suggested that replacing WPI with PPH at the ratio of 8/5 could be the optimal selection for composite gels. The findings indicated that PPH could be used as a substitute for whey protein to develop new products for different consumers. Composite gels could deliver nutrients such as vitamins and minerals to develop snack foods for elders and children.

## 1. Introduction

Oropharyngeal dysphagia (OD) is a common clinical symptom in the elderly [1]. It may lead to decreased appetite and even mental illness [2]. In order to avoid aspiration symptoms and to provide adequate nutrition, foods should be carefully chosen to satisfy the elderly with OD [3]. Texture-modified semi-solid foods with low strength, easy swallowing, and unique rheological properties have been used as the mainstream choices for OD patients [4]. The gelation of proteins could form a desirable structure for food products. The texture of protein gels could be regulated by changing the structure of the gel network. It could result in protein gels as a potential food for the elderly with OD [5].

Animal proteins are widely used in food gels, owing to their excellent gelatinization and nutritional value [6]. As processing animal proteins results in more carbon emissions, alternative proteins such as plant proteins have attracted more attention. Mixing plant and animal proteins could be used to develop food products with new gel structures [7]. Several plant proteins, such as soy protein [8], pea protein [9], and potato protein [10], have been used to partially replace whey protein to prepare composite gels. Composite protein gels could present denser microstructures and different mechanical properties when compared with single protein gels [11,12,13,14,15]. Although the displacement of animal protein with plant protein could decrease the hardness of gels, it could be a good choice for developing products for the elderly with OD [5]. Plant proteins usually exhibit low solubility. This could affect the interaction between plant proteins and animal proteins and have a negative effect on gel hardness. The formation of gels could require a higher protein content that supplies enough soluble protein. Instead, increasing the plant protein solubility could be another way to improve the properties of composite gels [16,17].

Enzymatic hydrolysis is an effective method for increasing the solubility of plant proteins [18]. The application of potato protein with low solubility has been limited because of its poor functionalities. Enzymatic hydrolysis of potato protein has been used to improve its solubility and the release of bioactive peptides. The potato protein hydrolysate was able to retard lipid oxidation of food products [14] and exhibited some biological activities, including regulating blood pressure and antioxidant effects [16,17]. Using PPH as an alternative protein for WPI was supposed to be a potential new strategy for developing composite plant and animal protein gels. However, little has been available to prepare composite plant and animal protein gels by mixing PPH and WPI. In comparison, soy protein hydrolysates have been reported to reduce the hardness of soy protein gels [19]. On the contrary, plant protein hydrolysates prepared by enzymatic hydrolysis comprise peptides and free amino acids. It has been demonstrated that amino acids could enhance protein gel properties [20]. Contradictory results indicated that the effect of PPH on the properties of WPI/PPH composite gels should be addressed.

When developing food for the elderly, the release of nutrients should be considered. In recent years, the INFOGEST static in vitro model [21] has been used to simulate the nutrients released, including protein factually. Based on this digestion model, Lorieau et al. [22] have demonstrated that the protein gel’s hydrolysis kinetics and amino acid release significantly differ from the protein solution. Protein gels with different microstructures and properties could result in different digestion behaviors [23]. The loose gel structure could produce higher pepsin diffusivity than the compact gel structure [24]. Gels with different structures could lead to a difference in enzymatic hydrolysis during digestion. The soy protein hydrolysate content was demonstrated to affect the properties of the hybrid gel prepared with soy protein hydrolysates and soy protein [19]. However, little has been conducted on the digestion of those composite gels.

This study aimed to develop gels that were easy to swallow with mixtures of WPI and PPH. The effect of different WPI/PPH ratios on the gelling properties of composite gels was demonstrated. Moreover, the International Organization for Standardization of Dysphagia Diet (IDDSI) was used to classify the swallowing levels of gels. Furthermore, the microstructure and digestive kinetics of composite gels were characterized.

## 2. Materials and Methods

### 2.1. Materials

Commercial whey protein with a protein content of 90.9 wt% was obtained from Hilmar Company (Hilmar, CA, USA). Commercial potato protein with a protein content of 62.5 wt% was purchased from Shanxi Ciyuan Biotechnology Co., Ltd. (Xi’an, China). Alcalase 2.4 L FG (2.4 Au) was purchased from Novozymes Biotechnology Co., Ltd. (Copenhagen, Denmark). Transglutaminase (120 U/g protein) was purchased from Jiangsu Yiming Biological Co., Ltd. (Taixing, China). Pepsin from porcine gastric mucosa (250 U/mg protein), pancreatin (8 × USP specifications), and fast green were purchased from Sigma Aldrich, Inc. (St. Louis, MO, USA). The other reagents were of analytical grade.

### 2.2. Preparation of Potato Protein Hydrolysate

Potato protein hydrolysate was prepared according to the method of Cheng et al. [25]. Alkaline protease was used to hydrolyze the potato protein (40 g/L). The ratio of the enzyme to the substrate was fixed at 1:100. After hydrolysis for 120 min at pH 8 (pH was adjusted using 1 M NaOH), the pH of PPH was adjusted to 7.0 using HCl (1 mol/L). The supernatant was collected and lyophilized. The protein content of PPH was 61.8 ± 0.36%.

### 2.3. Preparation of Composite Protein Gel

WPI was dissolved in distilled water and heated at 75 °C for 10 min, followed by dispersion of PPH by magnetic stirring. The solution of mixtures (with a protein concentration of 130 g/L) was prepared at different WPI and PPH ratios of 8/5, 9/4, 10/3, 11/2, 12/1, and 13/0. The TG enzyme (30 U/g protein) was placed into the mixture solution and the solution was kept at 50 °C for 1 h before gelling. The composite gel was formed by loading the solution inside glass tubes (16.5 cm i.d. × 50 cm height), then heating the solution for 10 min at 90 °C. Afterward, gel samples were placed in a refrigerator at 4 °C overnight for further use.

### 2.4. Dynamic Rheological Properties

Dynamic rheological measurements of the mixture solution were conducted using a Discovery HR-1 hybrid rheometer (TA Instruments, New Castle, DE, USA) according to the methods of Cheng et al. [26]. The standard concentric cylinder geometry, which included a cup with a radius of 15 mm and a DIN rotor that had a radius of 14 mm and a height of 42 mm, was used. The mixture solution (20 mL) was loaded into the cup and covered with a thin layer of silicone oil (1 mL) to prevent evaporation. The procedure was divided into four steps. The samples were kept at 50 °C for 1 h, followed by heating from 50 to 90 °C at a rate of 5 °C min^−1^. Then, the temperature of the samples was held at 90 °C for 10 min, followed by cooling the sample from 90 °C to 25 °C at a rate of 5 °C min^−1^.

### 2.5. Textural Profile Analysis (TPA)

The texture analyzer (Stable Micro Systems TA. XT plus, Surrey, UK) was used to assess the texture characteristics of the gels according to the methods of [27]. The probe of P0.5 was used. The test parameters were as follows: pre-test speed and test speed were 1 mm/s and 2 mm/s, respectively. The trigger force of 1 g was used. The compression deformation was 30% [28].

### 2.6. IDDSI Testing

According to the experimental method proposed by Cichero et al. [29] and the classification proposed by the International Organization for Standardization of Dysphagia Diet (IDDSI), a spoon test was used to confirm the food swallowing grade of WPI/PPH composite gels in the IDDSI framework. In the test, a spoon was used to scoop part of the food. Then, the spoon was tilted and the food fell under the action of gravity. The food should continue to hold its initial shape on a plate.

### 2.7. Water-Holding Capacity (WHC)

The WHC of gels was measured according to Mao et al. [30], with some modifications. In brief, gel samples were removed from the container and wrapped with double filter paper, followed by putting the gels into a 50 mL centrifuge tube. Cotton balls were loaded to the bottom of the centrifuge tube to remove water and to protect the gels from fracturing. The tubes were centrifuged at 2000× *g* for 10 min. The WHC was calculated using the formula as follows:

(1) $$\mathrm{WHC}\%=\frac{m_{2}}{m_{1}}\times 100\%$$

where *m*_1_ is the initial mass of gel; *m*_2_ is the mass of gel after centrifugation.

### 2.8. Fourier Transform Infrared Spectroscopy (FTIR)

The composite gel samples were cut into small cubic pieces and frozen at −20 °C for 24 h. The frozen gel samples were lyophilized in a lab-scale freeze dryer for 48 h. The FTIR spectra of lyophilized gel samples were recorded in the range of 4000–600 cm^−1^, with a resolution of 4 cm^−1^. The secondary structure of the protein in the gels was analyzed by the software of PeakFit (Nicolet iS50, Thermo Fisher, Waltham, MA, USA).

### 2.9. Chemical Forces in Protein Gels

The chemical forces in gels in the composite protein gels were estimated by the method of Jiang [31]. The gel sample (0.5 g) was dissolved with 4.5 mL of selected solvent with a thermal process at 80 °C for 30 min. The gel suspension was centrifuged at 5000× *g* for 15 min. The protein concentration in the supernatant was determined by biuret methods. Furthermore, the protein solubility in different solvents was used to demonstrate the main chemical forces in the gels.

Three solvents were used, including 50 mM sodium phosphate (pH 7.0): 8 M urea (to estimate hydrogen bond), 0.5% SDS (to estimate total noncovalent forces), and 0.25% β-mercaptoethanol (to estimate disulfide bonds).

### 2.10. Low-Field Nuclear Magnetic Resonance (LF-NMR)

According to the method of Yan et al. [32], the measurement of the transverse relaxation time (T2) was tested by a low-field pulsed NMR analyzer (NIMI20-030V-I, Suzhou Niumag Analytical Instrument Co., Suzhou, China). The composite gels were placed in a 40 mm NMR tube. The transverse relaxation time (T2) was obtained using a Carr–Purcell–Meiboom–Gill pulse sequence. MultiExp Inv Analysis Software was used for data analysis.

### 2.11. Confocal Laser Scanning Microscopy (CLSM)

Gel samples were primarily cut into slices of about 1 mm and stained with 2 μL fast green (10 mg/mL) for 30 min. The dyed gel slices were placed on a single concave 1.5 mm-thick slide (Sail Brand, Jinliu Instrument Co., Ltd., Nanjing, China) and covered with a cover slip. The microstructure of the composite gels was characterized by CLSM (TCS SP 5, Leica Microsystems Inc., Heidelberg, Germany) with a 40× magnification lens. The excitation line for protein was 633 nm [13].

### 2.12. Scanning Electron Microscopy (SEM)

The samples were cut into small pieces and fixed in 2.5% glutaraldehyde for at least six hours. After being rinsed thrice in 50 mM phosphate buffer solution (PBS) at pH 7.0, the samples were dehydrated with ethanol, followed by vacuum freeze-drying. Dried samples were sputter-coated with gold and microstructure images of the gels were captured by a scanning electron microscope (SEM) (HITACHI SU8010, Tokyo, Japan) at 8000 and 30,000× magnification [33].

### 2.13. Swelling Ratio

The swelling ratio was tested according to Ozel et al. [34]. Briefly, a gel sample (5 g) was first put into a zip-lock bag (perforated to allow water to pass through freely) and soaked in 50 mL of 10 mM PBS at pH 7.0 at room temperature. Then, the sample was taken out at different times (0.5, 1, 2, 3, 4, 5, and 6 h) and weighed after gently removing the excess water with filter paper. The swelling ratio was calculated by the formula as follows:

(2) $$Swelling\;ratio\%=\frac{M_{t}-M_{0}}{M_{0}}\times 100\%$$

where *M_t_* is the mass of gel tested at different times; *M*_0_ is the initial mass of gel.

The generalized semiempirical equation was used to describe the swelling kinetics [35].

(3) $$Kt^{n}=\frac{SR_{t}}{SR_{\infty }}$$

where *SR_t_* is the swelling ratio of the gel at different times; *SR*_∞_ is the swelling ratio of the gel after equilibrium; *K*, *t*, and n represent rate constant, swelling time, and release exponent, respectively.

### 2.14. In Vitro Digestion

In vitro digestion was conducted using the INFOGEST model [21]. During gastric digestion, the pH was kept at 3.0 with 1 M HCl; HCl consumption was recorded at different times (0.5, 1, and 2 h). During intestinal digestion, the pH was kept at 7.0 with 1 M NaOH; NaOH consumption was recorded at different times (0.5, 1, and 2 h) [13].

Gastric digesta (0.5 mL) and intestinal digesta (0.5 mL) were taken at different times (0, 30, 60, and 120 min), followed by inactivating the enzyme immediately at 95 °C for 5 min. Then, 200 μL of each digested sample was diluted and centrifuged for 10 min at 10,000× *g*. The supernatant was collected to determine the release of the free amino group by the OPA method [22].

### 2.15. Statistical Analysis

All trials were conducted with three replicates. Each replication was performed on different days with newly prepared PPH. Data were analyzed using SPSS (Statistical Analysis System, IBM Corp., Armonk, NY, USA) with one-way ANOVA. Significant differences in data (*p* < 0.05) were analyzed using Duncan’s multiple comparison analysis. Sigmaplot for Windows Version 10 (Systat Software Inc., Chicago, IL, USA) was used to plot the figures.

## 3. Results

### 3.1. Rheological Properties

**Figure 1:**
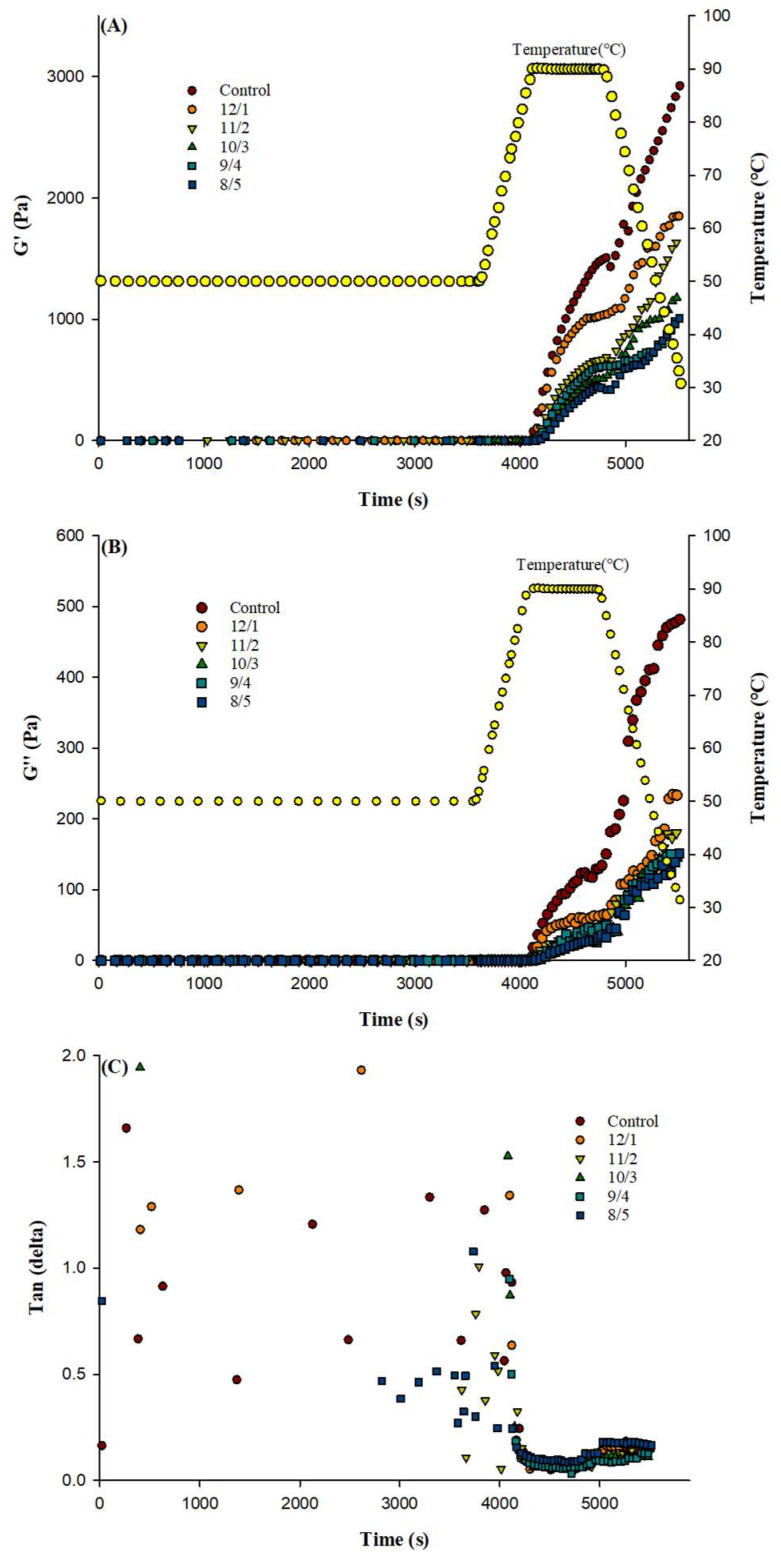
Storage modulus (G′) (**A**), loss modulus (G″) (**B**), and tanδ (**C**) of WPI/PPH composite gels at different ratios during gel formation.

Storage modulus (G′), loss modulus (G″), and tanδ of composite protein solutions during gel formation are shown in Figure 1A,B. With the increase in PPH ratio, the final G′ and G″ of the gels were significantly reduced, whereas the final tanδ of the gels increased. The final G′ of the composite gel with a WPI/PPH ratio of 8/5 had nearly a 3-fold reduction compared with the control, while the G″ of the composite gel with a WPI/PPH ratio of 8/5 decreased about 4-fold when compared with the control. It demonstrated that an increase in PPH ratio increased the tanδ value of the composite gels. This suggested that the substitution of WPI with PPH resulted in soft composite gels; a possible explanation would be that PPH occupied the position of WPI and weakened the hydrophobic interaction between the whey protein molecules, which might affect the formation of WPI aggregates during heating. The change rate of G′ and G″ during the heating step could be used to indicate the formation rate of WPI aggregates. It was evident that the change rate of G′ and G″ of composite gels with WPI/PPH of 13/0 was higher than that of 8/5.

### 3.2. Textural Profile Analysis

Hardness was one of the leading mechanical properties of the gel and could directly affect the taste of the food [36]. As shown in Table 1, hardness presented an upward tendency, with an increased WPI ratio. This was consistent with the results of the rheological analysis and was not surprising, because the concentration of WPI decreased. The hardness of composite gels with WPI/PPH of 13/0 was 2.38, 2.08, 1.82, 1.48, and 0.52 times higher than the samples with ratios of 8/5, 9/4, 10/3, 11/2, and 12/1 (*p* < 0.05). Amici et al. [37] reported similar results when they replaced whey protein with rapeseed protein. Chewiness was an additional parameter of hardness for solid gel samples [38]. The chewiness of composite gels with a WPI/PPH ratio of 13/0 was 1.60 times higher than that of 8/5, while the remaining samples showed little difference. Springiness could be used to characterize the recovery capability of the gel after compression under extrusion pressure [39]. Unlike the results of hardness, replacing WPI with PPH enhanced the springiness of gel samples. This was consistent with our hypothesis on the results of tanδ that a higher tanδ resulted in soft gels. Higher springiness was obtained in the gels with a WPI/PPH ratio of 11/2 and 10/3. The springiness of gels with a WPI/PPH ratio of 11/2 increased by 89.75% and, with a WPI/PPH ratio of 10/3, increased by 82.09% compared with the control samples (WPI/PPH ratio of 13/0). Wendin et al. [40] indicated that springiness was particularly important for swallowing difficult food. Therefore, the increment in the PPH ratio was conducive to the development of easy-to-swallow food, according to the conclusions drawn by Wada et al. [41] and Park et al. [42].

### 3.3. Appearance and IDDSI Testing

**Figure 2:**
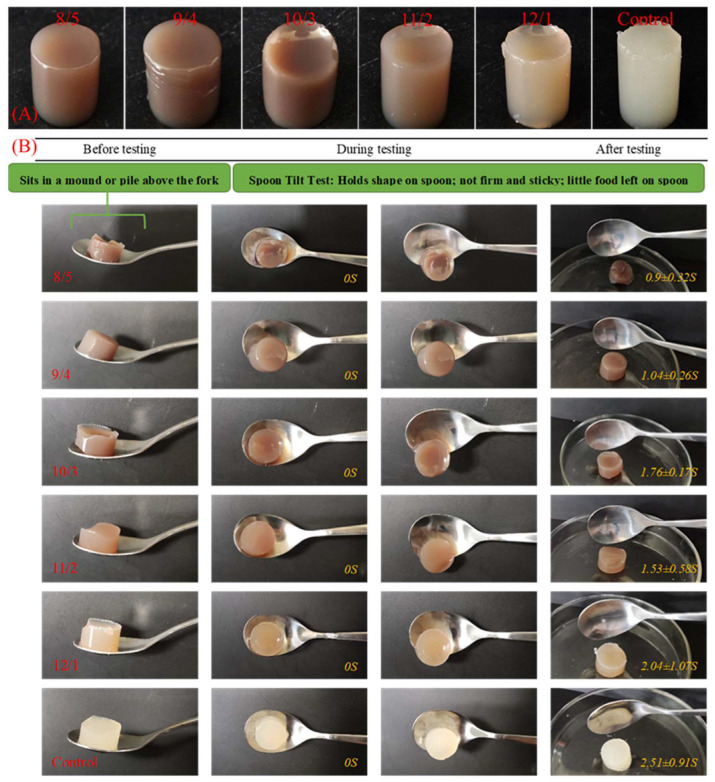
Photographs of composite gels prepared with WPI/PPH at different proportions (**A**) and spoon tilt test (**B**). In the spoon tilt test, four photographs were taken of each sample: when the sample was placed horizontally on the spoon (before testing); when the spoon was tilted; when the gel in the spoon slid (during testing); when the sample slid into the petri dish (after testing).

As shown in Figure 2A, WPI/PPH composite gels that were formed at different proportions were solid. The gels showed smooth and uniform surfaces in appearance. The diameter of the gels with higher WPH/PHH ratios (8/5 to 10/3) was more significant than the control, whereas their height was lower than the control. This suggested that partially replacing WPI with PPH led to the formation of soft gels. This was consistent with the results above.

According to the conclusions of Wada et al. [41], the suitable food bolus for swallowing was defined as a texture under 15,000 N/m^2^ in hardness. The gels prepared in the experiment conformed to this characteristic. To confirm this, researchers also needed to follow the IDDSI method [29] to evaluate the grade of composite WPI/PPH gels. For these samples, the spoon tilt test was selected for the IDDSI testing. The results are shown in Figure 2B. Samples of the same size were taken and placed in the spoon. The spoon was tilted at the same angle and the time was recorded as 0 s. The gels would slide into the container during this test.

Moreover, the time that the samples fell was recorded as the end time. According to the results, the gel samples were cohesive enough and could hold their shape on the spoon. When the spoon tilted, the gel samples in the spoon slipped in a short time. The gel samples slid off easily, leaving little gel on the spoon. This indicated that these gel samples could belong to level 4 foods in the IDDSI framework [29]. The gels with a WPI/PPH ratio of 8/5 demonstrated the least falling time, which could have been related to their low hardness and high springiness.

### 3.4. Water-Holding Capacity (WHC)

**Figure 3:**
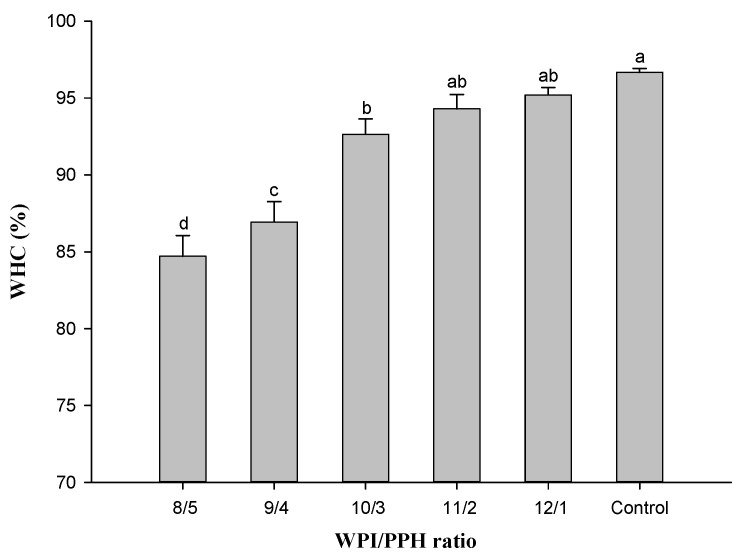
The water-holding capacity of composite gels at different WPI/PPH ratios. Different letters (a–d) mean significant differences (*p* < 0.05).

Water-holding capacity describes water entrapments in the network of gels and is closely associated with the rheological properties of gel products (hardness, elasticity, viscosity, and tenderness) [43]. As shown in Figure 3, replacing WPI with PPH could reduce the WHC of whey protein gels. The WHC of composite gels with a WPI/PPH ratio higher than 10/3 was lower than that of the control (*p* < 0.05). The WHC of composite gels with the WPI/PPH ratio of 8/5 was reduced by 12.37% compared with the control. The gels with higher G′ and hardness generally had larger WHC, since, under the same centrifugal force, the stiff gels would experience a lower extent of compression than the weaker ones; hence, less water was expelled from the former [15]. The possible reason could be that PPH could reduce the surface charge of whey protein molecules by TGase enzymatic crosslinking. In addition, Chu et al. [44] reported that the gel network structure was affected by WHC, which was also consistent with the microscopy images of the gel microstructure shown later.

### 3.5. FTIR

**Figure 4:**
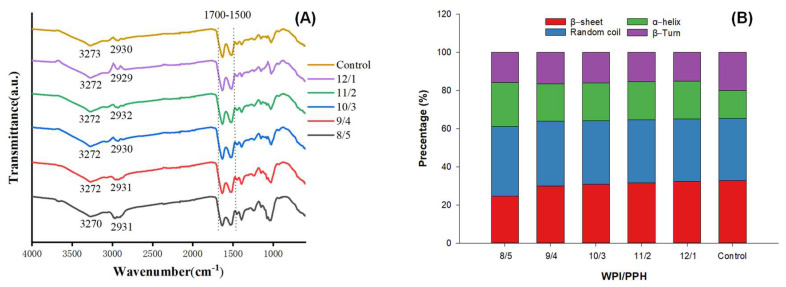
FTIR spectra (**A**) of WPI and PPH composite gels with different proportions (4000–600 cm^−1^). The secondary structure (**B**) content of protein in composite gels at different WPI/PPH ratios.

The FTIR spectra (Figure 4A) showed that adding PPH did not form new peaks in the composite gels. This suggested that WPI and PPH did not form new bonds. The absorption peaks observed at 3500–3200 cm^−1^ indicated the formation of hydrogen bonds [44]. The areas of these two peaks increased with the increment in the PPH ratio. This suggested that incorporating PPH into composite gels could increase the content of hydrogen bonds. The reason could be that PPH changed the gels’ secondary structure of whey protein. The peaks at 2950–2800 cm^−1^ were due to the C-H stretching in the CH2 and CH3 groups. The width of the peaks at 2950–2800 cm^−1^ was broadened as the ratio of PPH increased. This could be related to the interaction between the CH2 and CH3 groups. Inconsistently, Zhang et al. [45] thought the change could be the stretching of CH2 and NH_3_+.

The amide I band (1700–1600 cm^−1^) was the most sensitive region that could be used to interpret the protein secondary structure by multiple peak fitting analysis [46]. The shift of the amide I band was related to the change in the protein secondary structure [45]. The complex protein ratios significantly affected the secondary structure of proteins in the gel, as shown in Figure 4B and Table S1. Increasing the whey protein ratio resulted in higher contents of β-sheet and β-turn, which led to lower contents of α-helix and random coil. Gao et al. [47] showed that the content of β-sheet was positively correlated with the hardness of the gel, which was consistent with the results of this study. The content of α-helix in the gels with a WPI/PPH ratio of 8/5 was 0.56 times higher than that of 13/0 (control) (*p* < 0.05). It seemed that PPH hindered the unfolding of whey protein during thermal denaturation.

### 3.6. Forces in Protein Gels

**Figure 5:**
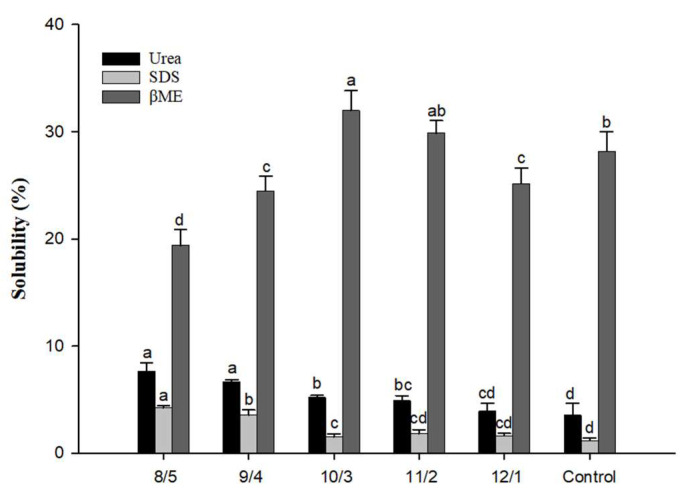
Solubility of composite gels with different WPI/PPH ratios in different solvents. Different letters (a–d) mean significant differences (*p* < 0.05).

The formation of protein gels was related to covalent and noncovalent forces involved in protein aggregation. The physicochemical bonds and interactions in protein gel could be analyzed using the solubility of gel in different force-disruption agents [31]. Figure 5 shows that the highest solubility of gel samples was obtained in β-mercaptoethanol. This indicated that the disulfide bond was the main force for forming protein aggregates in the gel structure. It suggested that the dominant network of composite gels comprised whey protein. The solubility of the gels with a WPI/PPH ratio of 8/5 in β-mercaptoethanol solution reduced by 31.17% when compared with the control. This was consistent with the report by Xia et al. [15], who made the gels by substituting whey protein isolate with soy protein isolate; the different ratios did not change the main function of the disulfide bond.

The solubility of protein gels in urea and SDS solution increased with the increment of the PPH ratio. Compared with the control group, the solubility of the gels with a WPI/PPH ratio of 8/5 in urea and SDS solution increased by 1.14 and 2.62 times, respectively. This indicated that a partial substitute of whey protein with PPH increased the content of the hydrogen bond. This was consistent with the result of the FTIR. Comparing gels with the WPI/PPH ratio of 8/5 with the control samples and lowering the content of the disulfide bond could indicate the decrease in gel strength [48]. In contrast, enhancing the content of noncovalent bonds, including hydrogen bonds, could be a sign of raised gel elasticity.

### 3.7. LF-NMR Analysis

**Figure 6:**
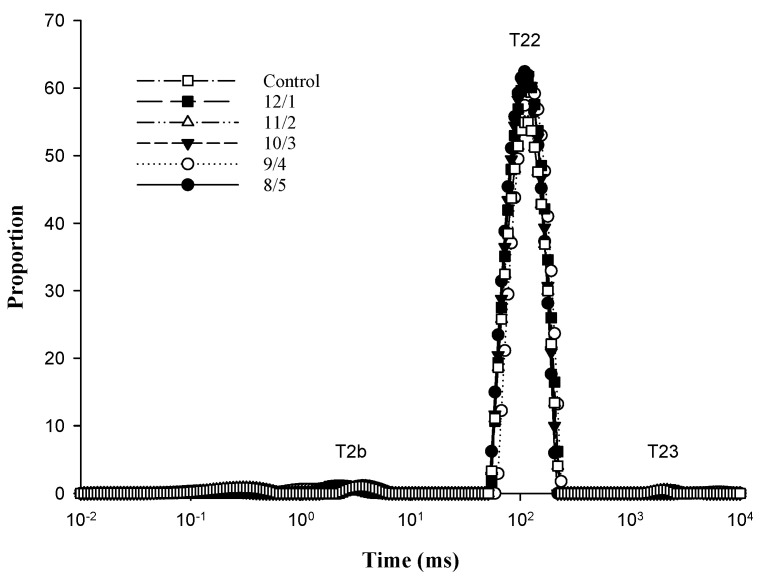
The water distribution of composite gels with different WPI/PPH ratios in LF-NMR. Different letters indicate that the results have significant differences (*p* < 0.05).

Gels on the distribution of T2 relaxation times and relaxation parameters for gels with different proportions of WPI and PPH are shown in Figure 6 and Table 2. Water existing in the composite gels was dominated by immobilized water according to the proportion of peak T22. As shown in Table 2, no significant difference in the proportion of bound water (T2b) and immobilized water (T22) among different samples was shown. The free water (T23) content increased significantly as the WPI ratio increased; the proportion of free water in the control group was 78.43% higher than the WPI/PPH ratio of 8/5. This indicated that the PPH ratio significantly affected T23, which could have resulted in the hydrogen bonds between WPI and PPH [49].

### 3.8. Microstructure of Composite Gels

**Figure 7:**
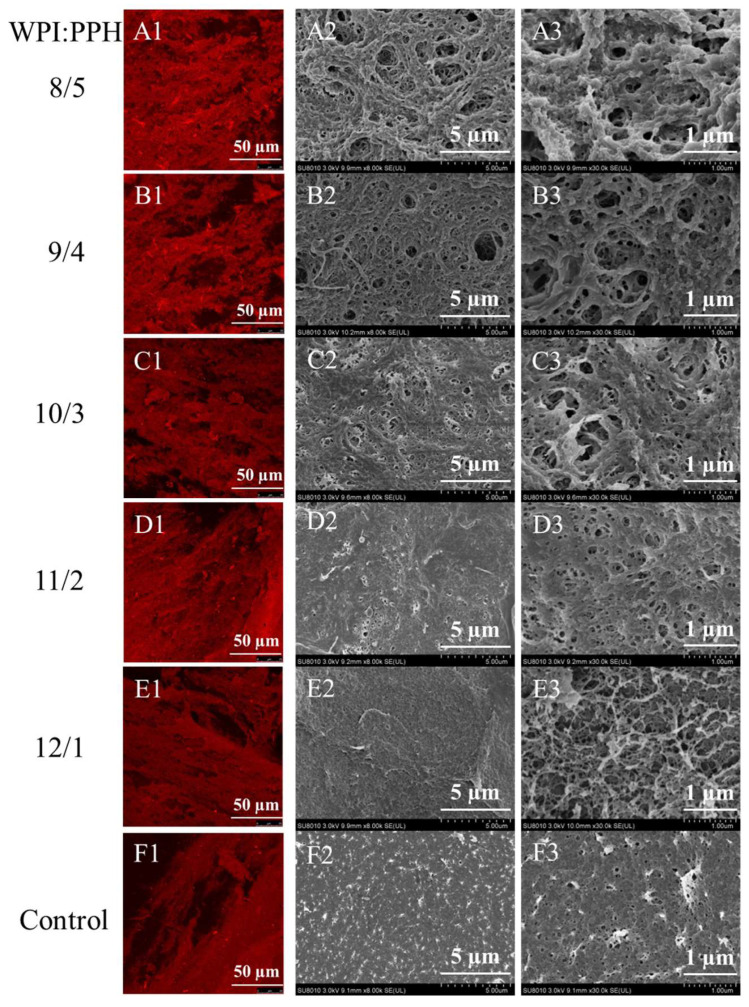
CLSM and SEM of WPI and PPH composite gels with different WPI/PPH proportions at 8/5 (**A**), 9/4 (**B**), 10/3 (**C**), 11/2 (**D**), 12/1 (**E**) and 13/0 (Control) (**F**).

Confocal laser scanning microscopy (CLSM) and SEM were used to observe the microstructure of composite WPI/PPH gels, as shown in Figure 7. As stable solid gels were formed, all the gel samples displayed continuous and intact protein networks in the gel matrices. The CLSM image showed that the gel with a WPI/PPH ratio of 13/0 (control) exhibited little gaps in the gel matrix. This indicated that a compact and fine protein network in the microstructure existed in the control sample. At the same time, incorporating PPH into composite gels led to gaps and evident protein aggregates in the gel matrix. A higher PPH ratio could result in gaps and aggregates with a larger size in the gel matrix. This indicated that loose and coarse protein networks in microstructure existed in the composite gels. This was no surprise, because the results of FTIR had suggested that PPH could prevent whey protein from unfolding; the protein molecules could keep a certain distance because of the steric hindrance effect.

Meanwhile, PPH-induced increments in chemical forces, including hydrophobic interaction and hydrogen bonds, could improve protein aggregation. Therefore, large aggregates were formed. The replacement of whey protein with PPH decreased the concentration of whey protein and its aggregates. This could lead to large gaps, because the density of the protein aggregates decreased and the water molecules could be filled into the blank space.

Compared with CLSM, SEM presented a higher magnification and more details in the microstructure could be observed. SEM images were captured by magnifications of 8000 (Figure 7A2–F2) and 30,000 times (Figure 7A3–F3). SEM images confirmed the microstructure characteristics of the gel samples interpreted from the CLSM images. All gels presented a porous network in the microstructure. The microstructure was well consistent with the hypothesis of gel formation from Mezzenga and Fischer [50]. Oligomers, primary aggregates, and large self-similar aggregates formed the final three-dimensional structure of the protein gel.

The images of the control group showed a protein network with a thin skeleton and a dense matrix in the microstructure. For composite WPI/PPH protein gels, protein networks with thick skeletons and coarse matrices in the microstructure were observed. Different WPI/PPH ratios led to microstructures with aggregates and mesh in different sizes. Increasing the WPI/PPH ratio from 12/1 to 8/5 enhanced the thickness of the skeleton in the protein network and enlarged the mesh size. The difference in the gel microstructure could well explain the mechanical properties of the composite gels. The control samples with compact and fine microstructures could exhibit higher hardness and storage moduli, while the low hardness and storage moduli of composite gels with a WPI/PPH ratio of 8/5 were related to their loose and coarse microstructure. In addition, according to the analysis of Joeres et al. [51], a denser gel structure led to a better water-holding capacity. This was consistent with the results of the WHC.

### 3.9. Proposed Composite Gel Forming Mechanism

**Figure 8:**
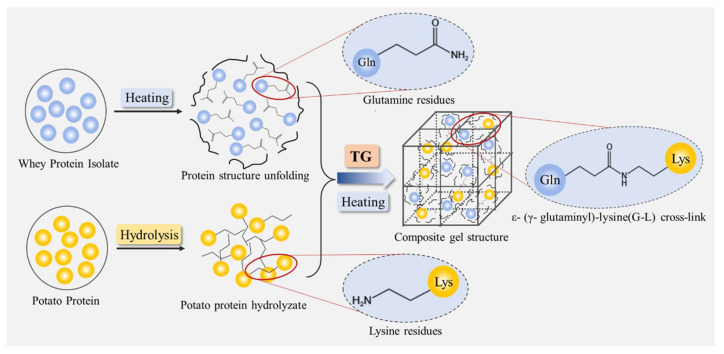
The schematic diagram of WPI/PPH composite gels.

Based on the above analysis, the possible gelling mechanism of the WPI/PPH composite gel was proposed (Figure 8). WPI was preheated at 75 °C for 10 min to unfold the protein. The exposed glutamine residues in the whey protein were crosslinked with lysine residues from potato protein hydrolysate by TG through covalent bonds. These covalent bonds could affect the aggregation of whey protein, followed by changing the microstructure of gels. The larger mesh size and thicker skeleton in the network in the microstructure of the composite gels led to lower hardness and larger springiness in the texture. Soft solid gels were formed.

### 3.10. Swelling Ratio

**Figure 9:**
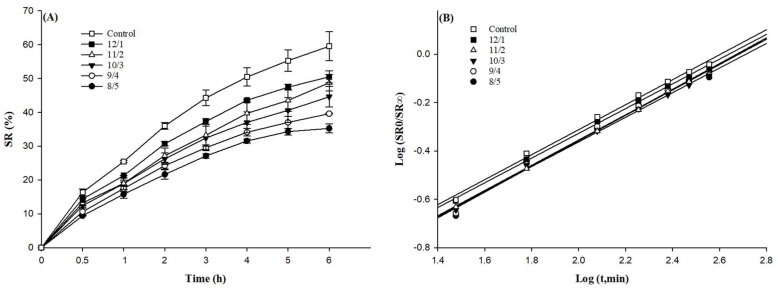
The swelling ratios (**A**) and the power law fitting curve of swelling kinetics (**B**) of composite gels with WPI and PPH at different proportions.

The swelling kinetics could be used to exhibit the ability of gel swelling in water [35]. It was determined by passively diffusing the solvent molecules into the gel network. As shown in Figure 9A, the swelling ratio of all samples increased during the swelling time of 6 h, indicating that solvent molecules could be absorbed and retained by the gels. The control sample had the highest swelling ratio of 59.53 ± 4.27%, while the samples with a WPI/PPH ratio of 8/5 had the lowest swelling ratio of 35.26 ± 1.30%. The swelling ratio was positively correlated with the water-holding capacity. The gels with denser networks displayed more substantial water-holding capacities and remarkable abilities to absorb solvent molecules. The tendency of the swelling result was also related to the gel strength, which was similar to the results of McCann et al. [52].

The generalized semiempirical equation used to describe the swelling kinetics was Kt^n^ = SR_t_/SR_∞_, converting the equation to LogK + not = Log(SR_t_/SR_∞_) [35]. When the sample was cylindrical, a perfect Fickian diffusion with an n value of 0.43, during which the rate of solvent penetration was slower (as it was the rate-determining step) than the chain relaxation rate. In the non-Fickian transport, with an *n* value of 0.43 < *n* < 1.0 during the transport system, the penetrant mobility and segmental relaxation rates were comparable [53]. The value of n for all gel samples was in the range of 0.43~1 (Table 3). This indicated that the mechanism of water diffusion in the gel matrix fitted the non-Fickian type of anomalous transport [54]. This suggested that the solvent diffusion in gels involved diffusion-controlled and swelling-controlled mass transfer characteristics.

### 3.11. In Vitro Digestion

**Figure 10:**
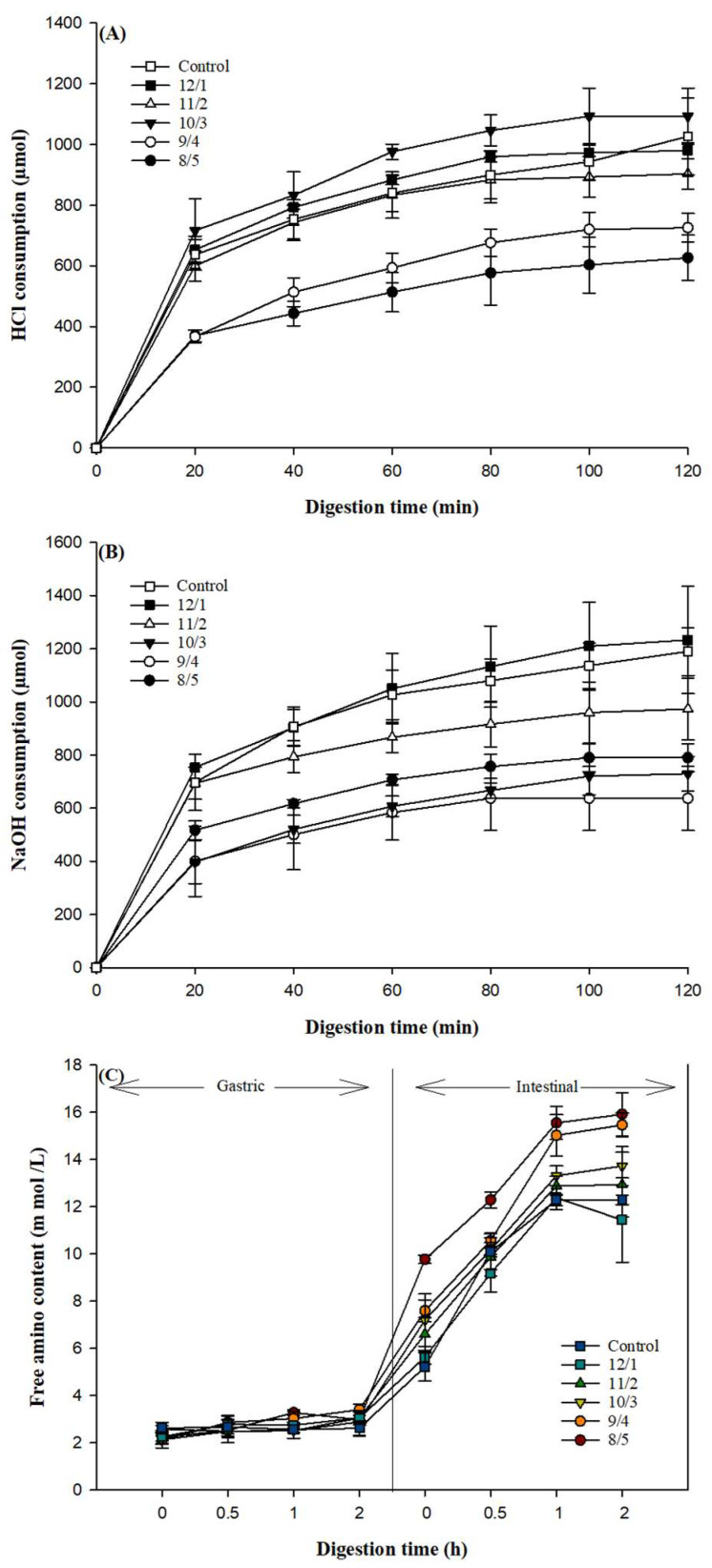
Acid (**A**) and base (**B**) consumption during gastric and intestinal digestion of composite proteins with different proportions. The amount of free amino groups released by composite gels during simulated gastrointestinal digestion (**C**).

The digestibility of nutrients was one of the essential parameters for evaluating the quality of the food. In vitro digestion of composite gels during the gastric and intestinal stage was demonstrated by consuming HCl and NaOH, as shown in Figure 10A,B. SEM and CLSM results showed that gel samples with a high WPI ratio displayed a dense microstructure, which consumed more HCl and NaOH during digestion.

The curves of the HCl consumption against digestion time were fitted to the first-order kinetic model y = y_0_ + a (1 − e^−kt^). It could be obtained that the values of the reaction rate constant k for gels with different WPI/PPH proportions of 8/5, 9/4, 10/3, 11/2, 12/1, and 13/0 were 0.0378, 0.0306, 0.0463, 0.0513, 0.0504, and 0.0457 (R^2^ > 0.92), respectively. The results showed that the replacement of whey protein with a low PPH ratio increased k, compared with the control, while the replacement of whey protein with a high PPH ratio reduced k, compared with the control. The reasons could be because the digestion of gels was influenced by various factors, including structural characteristics [55], interactions with other components, and methods of gel formation [56]. It has been reported that protein gels with dense microstructures and low porosity would exhibit a slow diffusion rate of digestive enzymes in a gel matrix [24]. The difference in the pore size of the gap in the gel network and the size of pepsin could affect the attack mode of enzyme on gels. When the first-order kinetic model was used to describe the reaction kinetics of the NaOH consumption, the reaction rate constant k for gels with different proportions (PPH/WPI = 8/5, 9/4, 10/3, 11/2, 12/1, and 13/0) were 0.0473, 0.0338, 0.0338, 0.0595, 0.0410, and 0.0419 (R^2^ > 0.98). The above results indicated that the composite gel with different ratios of PPH/WPI exhibited different digestion behaviors. Several studies have reported that the rate of gel digestion was affected by the microstructure [13,30,57]. When the enzyme reached the surfaces of protein aggregates, the gel network in a particular ratio improved the enzymatic hydrolysis of the gel.

Figure 10C shows the release of free amino groups during gel digestion. The free amino concentration of all gels rarely increased during gastric digestion. This suggested that the consumption of HCl might be mainly due to the diffusion of H^+^ into the swelling gel matrix. It was evident that large particles remained in the digesta. Differently, in the gastric stage, the concentration of the free amino group increased rapidly during intestinal digestion. Unsurprisingly, the NaOH consumption trend during digestion was not consistent with that of the free amino group release because a part of the HCl/NaOH consumed was transported into the gap in the gel matrix during digestion. The higher swelling ratio led to more diffusion flux of NaOH. The gel with a higher ratio of WPI consumed a higher amount of NaOH. In comparison, trypsin diffused slowly because of the tiny porosity. The gel with a higher ratio of WPI could release a less free amino group. The highest release of free amino groups was observed in gels with a WPI/PPH ratio of 8/5. This could be due to the presence of PPH, which was prone to be degraded by the enzyme. Furthermore, the coarse microstructure of gels with the WPI/PPH ratio of 8/5 could benefit the enzymatic hydrolysis of whey protein aggregates in the gel matrix.

## 4. Conclusions

In summary, it was demonstrated that WPI/PPH ratios significantly influenced the composite gels’ characteristics. An increment in the PPH ratio reduced the final G′, G″, hardness, and WHC of composite gels, while the springiness was enhanced. These properties were beneficial for the design of food that was easy to swallow. The composite gels were presented as level 4 foods in the IDDSI framework. They could be potential foods for patients with oropharyngeal dysphagia. Replacing the WPI with PPH could lead to the formation of coarse microstructures with larger pore sizes and thicker skeletons in composite gels. Composite gels with a higher ratio of PPH exhibited a lower swelling ratio. This could be evidence that the acid or base had a lower diffusion rate in these samples. Composite gels with higher PPH ratios displayed coarse microstructures in the gel matrix. The larger gap in the matrix made it easier for the trypsin to diffuse and attack the substrate. Therefore, gels with a higher PPH ratio demonstrated a higher rate and amount of amino acid release. Our results showed that replacing WPI with PPH could affect composite gels’ physical properties, microstructures, and digestion. The composite gels prepared with WPI/PPH at the ratio of 8/5 showed better mechanical and digestive characteristics. These findings suggested that the WPI/PPH composite gels could be used as a potential product for people with dysphagia and weak digestive ability. The potential bioactivity, such as the antioxidant properties of the gastrointestinal digesta, could be evaluated in future work. To know the nutritional properties of WPI/PPH composite gels, the determination of free amino acid release in the final gastrointestinal digesta is necessary.

## Figures and Tables

**Table 1:** The textural properties of the PPH and WPI composite gels. Different letters (a–e) in each column indicate significant differences (*p* < 0.05).

Sample	Hardness (g)	Resilience (%)	Cohesiveness	Springiness	Chewiness
8/5	23.89 ± 1.09 e	73.87 ± 0.94 c	0.97 ± 0.01 a	130.37 ± 15.7 c	29.29 ± 4.62 c
9/4	26.82 ± 0.16 d	78.35 ± 0.4 b	0.95 ± 0.01 ab	154.52 ± 14.92 b	42.52 ± 1.7 b
10/3	28.64 ± 0.54 d	77.53 ± 0.65 b	0.95 ± 0.01 ab	174.93 ± 10.78 ab	42.44 ± 5.71 b
11/2	32.58 ± 0.84 c	77.94 ± 0.71 b	0.96 ± 0.01 a	182.29 ± 1.31 a	47.50 ± 5.36 b
12/1	53.26 ± 1.08 b	77.94 ± 0.44 b	0.95 ± 0.01 ab	97.2 ± 0.13 d	49.61 ± 1.46 b
Control	80.87 ± 0.6 a	81.73 ± 0.51 a	0.93 ± 0.01 b	96.07 ± 2.2 d	76.30 ± 3.01 a

**Table 2:** Relaxation parameters of the PPH and WPI composite gels. Different letters (a–b) in each column indicate statistically significant differences (*p* < 0.05).

Sample	T2b (%)	T22 (%)	T23 (%)
8/5	2.65 ± 0.81	97.23 ± 0.72	0.11 ± 0.10 b
9/4	2.84 ± 0.41	97.06 ± 0.39	0.10 ± 0.07 b
10/3	2.64 ± 0.48	97.18 ± 0.47	0.19 ± 0.02 b
11/2	2.74 ± 0.28	97.06 ± 0.33	0.20 ± 0.06 b
12/1	2.06 ± 0.16	97.48 ± 0.20	0.46 ± 0.08 a
Control	2.86 ± 0.52	97.11 ± 0.49	0.51 ± 0.15 a

**Table 3:** The characteristic parameters of the power law model (Kt^n^ = SR_t_/SR_∞_) used to describe solvent diffusion in composite gels with WPI and PPH at different proportions.

WPI/PPH	8/5	9/4	10/3	11/2	12/1	Control
logK	−1.4219	−1.3990	−1.3816	−1.4080	−1.3509	−1.3459
n	0.5322	0.5223	0.5096	0.5252	0.5120	0.5172

## Data Availability

Data is contained within the article or supplementary materials.

## Supplementary Materials

The following supporting information can be downloaded at: https://www.mdpi.com/article/10.3390/foods12102040/s1, Table S1 Comparison of protein secondary structures determined by Fourier transform infrared (FTIR) with different ratios.
