# Supplementary material for: Mechanical Properties, Microstructure, and In Vitro Digestion of Transglutaminase-Crosslinked Whey Protein and Potato Protein Hydrolysate Composite Gels
Source: Foods. 2023 May 18;12(10):2040. doi: 10.3390/foods12102040 (PMC10217323; doi:10.3390/foods12102040)
Supplement: Supplementary file 1 [file foods-12-02040-s001.zip › foods-2378554-supplementary.pdf]

foods

**Supplementary Materials:**

**Table. S1** Comparison of protein secondary structures determined by Fourier transform infrared (FTIR) with different ratios. Means with different letters (a-f) differ significantly ($p < 0.05$) among the data in the same row.

| | Ingredient (% w/w) | | | | | |
|---|---|---|---|---|---|---|
| | **8/5** | **9/4** | **10/3** | **11/2** | **12/1** | **Control** |
| **β-sheet** | 24.7±0.03f | 29.96±0.21e | 30.83±0.21d | 31.7±0.16c | 32.26±0.23b | 32.78±0.11a |
| **Random coil** | 36.42±0.37a | 33.93±0.17b | 33.43±0.26c | 33.02±0.08cd | 32.79±0.06de | 32.46±0.03e |
| **α-helix** | 23.06±0.07a | 19.51±0.16c | 19.67±0.17bc | 19.83±0.09b | 19.89±0.04b | 14.74±0.05d |
| **β-turn** | 15.82±0.41cd | 16.6±0.13b | 16.07±0.21bc | 15.45±0.18de | 15.06±0.27e | 20.02±0.15a |
